# Moulding the mould: understanding and reprogramming filamentous fungal growth and morphogenesis for next generation cell factories

**DOI:** 10.1186/s13068-019-1400-4

**Published:** 2019-04-02

**Authors:** Timothy C. Cairns, Xiaomei Zheng, Ping Zheng, Jibin Sun, Vera Meyer

**Affiliations:** 1 Tianjin Institute of Industrial Biotechnology, Chinese Academy of Sciences, Tianjin, 300308 China; 2 Key Laboratory of Systems Microbial Biotechnology, Chinese Academy of Sciences, Tianjin, 300308 People’s Republic of China; 3 Department of Applied and Molecular Microbiology, Institute of Biotechnology, Technische Universität Berlin, 13355 Berlin, Germany

**Keywords:** Cell factory, Filamentous fungi, *Aspergillus niger*, Systems biology, Synthetic biology, Protein secretion, Secondary metabolite, Citric acid

## Abstract

Filamentous fungi are harnessed as cell factories for the production of a diverse range of organic acids, proteins, and secondary metabolites. Growth and morphology have critical implications for product titres in both submerged and solid-state fermentations. Recent advances in systems-level understanding of the filamentous lifestyle and development of sophisticated synthetic biological tools for controlled manipulation of fungal genomes now allow rational strain development programs based on data-driven decision making. In this review, we focus on *Aspergillus* spp. and other industrially utilised fungi to summarise recent insights into the multifaceted and dynamic relationship between filamentous growth and product titres from genetic, metabolic, modelling, subcellular, macromorphological and process engineering perspectives. Current progress and knowledge gaps with regard to mechanistic understanding of product secretion and export from the fungal cell are discussed. We highlight possible strategies for unlocking lead genes for rational strain optimizations based on omics data, and discuss how targeted genetic manipulation of these candidates can be used to optimise fungal morphology for improved performance. Additionally, fungal signalling cascades are introduced as critical processes that can be genetically targeted to control growth and morphology during biotechnological applications. Finally, we review progress in the field of synthetic biology towards chassis cells and minimal genomes, which will eventually enable highly programmable filamentous growth and diversified production capabilities. Ultimately, these advances will not only expand the fungal biotechnology portfolio but will also significantly contribute to a sustainable bio-economy.

## The extensive product portfolio of filamentous fungi

Filamentous fungal cell factories are used by industrial biotechnologists to produce megatonnes of useful molecules worth several billion dollars per year [1]. The product portfolio of fungi is diverse and includes proteins, enzymes, secondary metabolites and organic acids. Fungal biomass is also a valuable product for food and textile industries, and most recently as a potential material in building construction (Table 1). Important enzymes derived from fungi include glucoamylases, phytases, pectinases, catalases, proteases, and glucosidases, with applications in food, textile, recycling, and other industries [2]. As just one example, fungal cellulase, hemicellulase, and ligninase cocktails are used to convert waste lignocellulosic biomass to fermentable sugars used as substrates for the biotechnological production of biofuels, with an estimated value of over €4 billion in 2016 [1]. Alternatively, fungal secondary metabolism is of significant pharmaceutical relevance, whereby low-molecular weight, structurally diverse nonribosomal peptides, polyketides, or terpenes are isolated for their potent bioactivities, including immunosuppressive compounds (e.g. cyclosporine A), cholesterol-reducing agents (e.g. lovastatin) and antibiotics (e.g. penicillin) [3]. In addition to these globally used drugs, the potential for discovery of new mode-of-action therapeutics from fungal secondary metabolism is promising, as over 60% of existing medicines are derived from natural products [4], and new techniques for activating the biosynthesis of these molecules in laboratory and pilot fermentation studies have recently been developed [5, 6]. Furthermore, various acids are produced by fungi, including gluconic, malic, itaconic, lactic, and fumaric acids (Table 1 and [7]). The most important acid is, arguably, citric acid, predominantly produced by *Aspergillus niger*, for use in food, pharmaceutical, and other industries, with an estimated global market value of over $2 billion [8, 9].

**Table 1:** Diversity of the fungal product portfolio

Fungus	Exemplar industrially relevant product(s)	Exemplar companies
*Aspergillus niger*	Enzymes (glucoamylases, proteases, glucosidases, catalases, phytases, pectinases) Organic acids (e.g. citric acid, succinate) Secondary metabolites	DSM (The Netherlands), Zymergen (USA), Novozymes A/S (Denmark) COFCO, Ensign, RZBC Group, (People’s Republic of China)
*Aspergillus oryzae*	Enzymes (amylases)	Novozymes A/S (Denmark), Amano Enzyme Inc. (Japan), Gekkeikan Sake Company Ltd. (Japan)
*Aspergillus terreus*	Enzymes (xylanases) Organic acids (itaconic acid) Secondary metabolites (lovastatin)	Merck (USA), Pfizer (USA)
*Acremonium chrysogenum*	β-lactam antibiotics (cephalosporins)	DSM (The Netherlands), Sandoz (Austria), Astellas Pharma (Japan)
*Blakeslea trispora*	Vitamins (β-carotene)	Universal Foods Corporation (Japan), DSM (The Netherlands), Pharmacia & Upjohn (USA)
*Fusarium venenatum*	Mycoprotein	Quorn Foods (United Kingdom)
*Ganoderma lucidum*	Composite materials (construction material) Imitation leather	Mycoworks (USA)
*Thermotelomyces thermophila*	Enzymes (cellulases, phytases, laccases)	Novozymes A/S (Denmark), BASF SE (Germany)
*Penicillium chrysogenum*	β-lactam antibiotics (penicillins) Enzymes (glucose oxidase)	DSM (The Netherlands), Pharmacia & Upjohn (USA), Merck (USA)
*Pleurotus ostreatus*	Composite materials (packaging material, construction material)	Ecovative (USA)
*Schizophyllum commune*	Textiles	MycoTEX (The Netherlands)
*Trichoderma reesei*	Enzymes (cellulases and hemi-cellulases)	DuPont (USA), Iogen Energy Corporation (Canada) AB Enzymes (Germany) Roal Oy (Finland)
*Ustilago maydis*	Itaconic acid	Toray Industries (USA), BASF SE (Germany)

Listed are fungal cell factories, key product(s), and companies that use each organism. Exemplar companies are meant only as a guide and were identified from published literature or patent databases. Note that this list is not exhaustive—for more examples, see [2]

With regard to use of filamentous fungi as a human food source, the global mushroom market value for champignons, shiitake, oyster, and others is expected to exceed $50 billion by 2022 [10]. In addition, mycoprotein produced by the ascomycete *Fusarium venenatum*, first marketed in 1984 as Quorn™, was recently valued at over $800 million worldwide [11]. In other applications, recent proof-of-principle experiments have demonstrated fungal biomass as a promising replacement for petroleum-based plastics or raw material in the textile and construction industry [12, 13]. Thus, the filamentous fungal product portfolio is undoubtedly extensive (Table 1) and is likely to expand to meet the needs of an emerging global bio-economy, a circular economy, and advances in healthcare [1, 14].

## Filamentous growth: the dynamic hypha

**Fig. 1:**
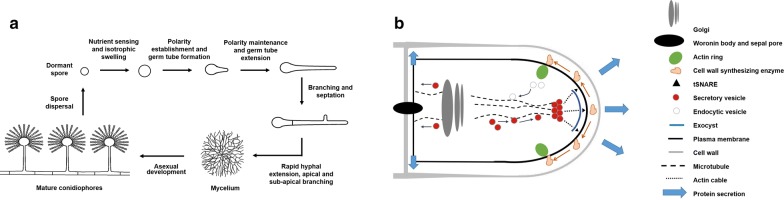
Schematic representation of filamentous fungal growth at cellular and subcellular levels. **a** Generic development of filamentous growth based on *Aspergillus* spp. Spores break dormancy and swell during a period of hydration and isotrophic growth, after which polarity is generated and maintained to form a germ tube. Hyphal extension results in branching at apical (tip) and subapical (intercalary) regions. Continued branching forms a network of hyphae termed a mycelium, and conidiophores are generated which bear asexual spores. Note that precise morphology and development will be different between industrially used fungal strains/species, and this is intended as a guide. **b** Depiction of subcellular organelles, cytoskeleton, and processes that couple growth and protein secretion at the hyphal tip. For explanation, see main text. Proteins are secreted predominantly at the tip; however, septal secretion has also been proposed

Technological advances in DNA sequencing and dedicated projects from the academic and industrial members of the fungal community have delivered a drastic increase in the number of annotated, curated, publicly available genomes for industrially important filamentous fungi, including the Ascomycetes *Aspergillus* spp., *Trichoderma* spp., *Penicillium* spp., and *Myceliophthora thermophila*, Basidiomycetes *Ustilago maydis* and *Ganoderma lucidum*, and Zygomycetes *Rhizopus* spp., amongst many others [15–17]. Despite these new resources, filamentous growth is a critical aspect of fungal biology that is not yet comprehensively understood [1]. Indeed, filamentous fungi have highly complex morphogenetic and developmental programs, which have been extensively studied in various model and industrially relevant Ascomycetes (e.g. *Aspergillus nidulans* and *A. niger*). In brief, under favourable nutritional conditions, spores break metabolic dormancy and undergo a period of isotropic swelling as water enters the cell (Fig. 1a). Next, myosins and formins recruit the actin cytoskeleton at a specific site to establish polarity, which is continually maintained to generate a highly polar germ tube [18, 19]. Growth of this cell occurs via extension at the apex, with membrane, extracellular hydrolytic enzymes, and cell wall synthesising proteins packaged into vesicles at the Golgi, and delivered along microtubule and actin cytoskeleton to the tip [20–23] (Fig. 1b). Vesicles aggregate at the tip at a site called the Spitzenkörper, after which they are tethered to the cell membrane via a multiprotein complex called the exocyst [20], with hyphal polarity mediated by various cell end marker proteins at the plasma membrane [21]. Exocytosis at the apex results in the insertion of new membrane, which is balanced by endocytic uptake of both membrane-bound or soluble material into the cell which occurs at a subapical actin ring (Fig. 1b) [23]. Moreover, polar cell extension occurs in an oscillatory manner [22], with pulses of influx of Ca^2+^ co-ordinating sequential rounds of actin polymerisation, exocytosis, and tip extension (for a recent review, see [19]). Internal turgor pressure is essential for continued growth and, as such, hyphal extension causes physical pressure on the established and newly synthesised cell wall. Consequently, control of cell wall integrity is a fundamental aspect of hyphal growth and viability [24]. Moreover, delivery of cell wall synthesising enzymes to the tip via vesicles must be balanced with the necessity to secrete extracellular enzymes for nutrient acquisition. Thus, secretion and cell wall growth are tightly linked, and genetic or abiotic perturbation to either process likely has significant impacts on the other.

As growth continues, hyphae divide by forming cross-walls known as septa, which contain pores for transfer of cellular content between cells, and a ‘plug’, termed a Woronin body, that can be used to block this pore [25]. Hyphae either branch at the tip (apical) or intercalary regions (subapical branching), and individual hyphae are able to fuse by a process termed anastomosis [26] to eventually generate a network of cells termed a mycelium (Fig. 1a). As the mycelium matures, secondary cell wall thickening occurs, and asexual development generates structures termed conidiophores, which bear spores that are essential for dispersal in the natural niche [27], but are generally considered to play a minor role in producing useful molecules. These dynamic morphological changes have critical implications for growth during solid-state fermentation, rheological aspects of submerged cultivation, and ultimately product titres.

## Why growth and morphology matter: a focus on submerged culture

**Fig. 2:**
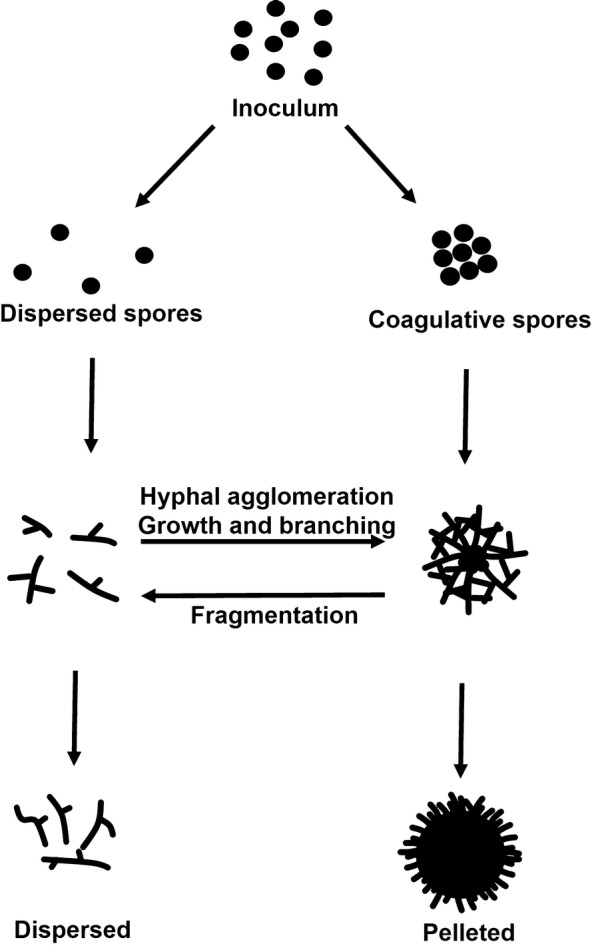
Schematic depiction of the formation of pelleted or dispersed macromorphological units during submerged cultivation. An inoculum of spores (black circles) either disperse or coagulate, which is dependent on fungal species and cultivation conditions (see main text). If dispersed, germinated hyphae can either agglomerate to form pellets or remain dispersed throughout cultivation. Sheering at pellet peripheries generates dispersed hyphal fragments in a phenomenon known as reseeding

A significant body of work over the last 30 years has interrogated the relationship between pellet morphology and product formation during liquid culture [28, 29]. In submerged fermentation, mycelia form various macromorphologies, resulting in dispersed hyphae, compact pellets, or intermediates of these growth types named loose clumps. These result from various interaction phenomena on spore and mycelial level in moist substrates. Pellet formation is conventionally distinguished by either coagulative or non-coagulative processes [29] (Fig. 2). Coagulative formation is representative for *A. niger* spores, which agglomerate after inoculation of growth media due to electrostatic and salt bridging between surface polysaccharides [29]. Additionally, hydrophobicity of spore surface proteins aids agglomeration, leading to germination of multiple physically grouped spores that form a single pellet [30, 31]. During non-coagulative pellet formation, e.g. as described for *Rhizopus* *oryzae*, spores remain dispersed during germination and agglomeration occurs at latter growth phases between branched hypha and consequently a single spore can potentially form a single pellet [28]. Some fungi, including *P. chrysogenum*, display characteristics of both the coagulative and non-coagulative types [29]. In this case, the agglomeration of different hyphal elements leads to hyphal clumps which can agglomerate to pellets [29]. Notably, fungal spores of the coagulative type can also form pellets in a non-coagulative way under specific media conditions, e.g. elevated pH [29]. The formation of loose clumps is less comprehensively understood, but presumably occurs in culture conditions which disrupt or inhibit agglomeration (see below).

Several advantages and disadvantages of either pelleted or dispersed macromorphologies are apparent. Firstly, pellets display enhanced resistance to sheer stress and minimum viscosity of bioreactor media [32–36]. However, internal areas of large pellets have low growth and metabolism due to poor oxygen diffusion, which may limit product formation [37]. In contrast, dispersed morphologies rapidly grow and do not have limitations in transport of nutrients [29, 36, 38]. The drawback to the dispersed growth state is a higher medium viscosity, limitations in gas–liquid mass transfer, and elevated susceptibility to sheer stress when compared to pellets [29, 36].

**Fig. 3:**
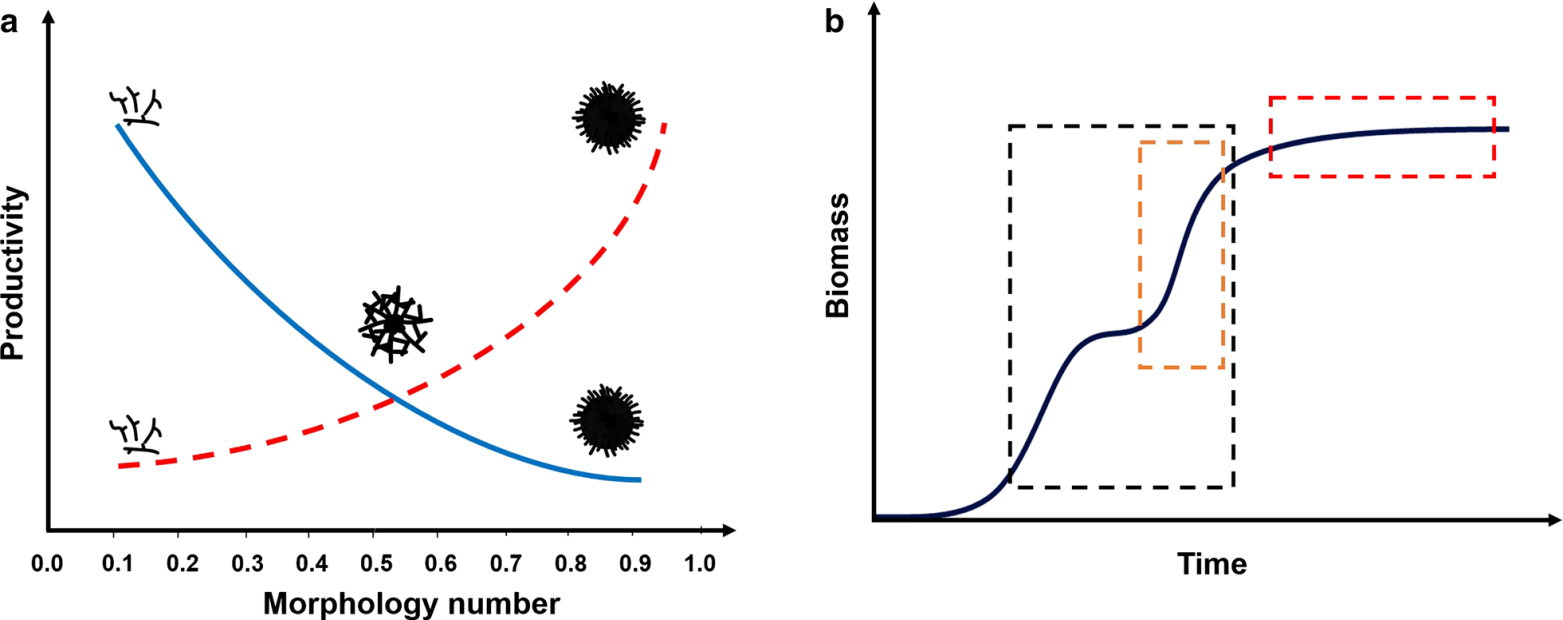
Product formation is dependent on fungal macromorphology and growth stage. **a** A schematic representation of pellet macromorphology which can be assessed by a dimensionless morphology number (MN) [45]. MNs varies between 0 (a one dimensional line) and 1 (a perfect circle). Fructofuranosidase and glucoamylase production by *A. niger* has been negatively correlated with an increasing MN (blue line) [45]; hence, these proteins are efficiently produced by dispersed mycelia. A hypothetical correlation between MN number and productivity is proposed for citric acid and secondary metabolites (red line). **b** Production of various classes of useful fungal molecules is also dependent on culture growth phase. The blue line depicts biomass accumulation in a hypothetical batch fermentation experiment. Protein and acid synthesis occurs during periods of exponential growth (black box). Formation of some acids occurs following a diauxic shift (orange box), for example citric acid fermentation in *A. niger* (see main text). Production of most secondary metabolites occurs during periods of low or zero growth (red box)

Significant efforts to optimise culture conditions to control fungal growth and morphology during industrial applications have thus been invested [29]. Fundamental factors affecting macromorphology and growth include carbon source/concentration [39], ion content (notably manganese) [40, 41], pH [29], density of spore inoculum [38], addition of surfactants [42], oxygen enrichment [43], agitation [44], osmolarity [45], addition of insoluble microparticles [46], or change from stirred tank to wave-mixed bioreactor equipment [36], amongst others. It is now possible to quantitatively measure effects of culture conditions on pellet morphology, specifically using particle parameters (e.g. projected area, circularity, aspect ratio, surface roughness) to generate a dimensionless morphology number for individual or groups of pellets [45] (Fig. 3a). Interestingly, the mechanistic basis for the formation of pellets or dispersed mycelia is increasingly described and explainable from hyphal extension rate, pellet fragmentation rate, and other bioreactor parameters using modelling approaches [34]. As these models are continually refined, they can be integrated with existing experimental evidence to refine and predict the underlying abiotic approaches that will enable a desired growth phenotype [47].

### Modelling fungal morphologies: from growth kinetics towards the subcellular

Modelling of fungal growth and morphology has predominantly focused on submerged culture and has advanced from preliminary models of growth kinetics to recent organelle and cytoskeletal-level frameworks of the filamentous lifestyle. Initial models developed in the 1960s were interested in pellet formation and growth, and assumed pellets consisted of hyphae growing radially outwards from a common centre, with all pellets of equal size in a bioreactor [48]. This enabled early models of various aspects of submerged culture. For example, Pirt tested the hypothesis that growth occurs at the outer pellet surface, with an inner mass of non-growing mycelium where nutrients are unable to diffuse [48]. Diffusion rate calculations revealed that the most important limiting factor that determines the thickness of the outer growing pellet layer is oxygen, which had critical implications for growth kinetics [48]. Indeed, the maximum critical transport distance for oxygen penetrating *A. niger* pellets has been experimentally confirmed as 200 µm, and internal areas beyond this limit are likely hypoxic [37]. Subsequent refinement of Pirt’s model introduced the reseeding phenomenon, whereby fragmentation at the pellet exterior results in the formation of smaller pellets and dispersed growth [49]. Consequently, these updated models assumed that pellet formation and size are dependent both on average hyphal growth rate and degree of fragmentation. The reseeding phenomenon required additional modelling of medium viscosity, fluid velocity, and sheer stress, amongst other parameters to better understand and predict growth in submerged culture [49].

More recent modelling of hyphal growth has gone beyond colony macromorphology to generate modelling frameworks based on distribution of intracellular components and organelles [34]. In this approach, intrahyphal passive transport from turgor pressure and active transport processes result in spatial distribution of organelles and other cellular components within individual hyphae, for example at the growing tip. The subcellular model developed by King [34] thus places critical emphasis on branch rate, whereby addition of new septa and apices occurs as a function of time and space within a growing hypha. Therefore, quantitative assessment of individual hyphal growth and prediction of macromorphological development from single spores will, therefore, only be possible when both space- and time-dependent processes are considered. Additionally, future modelling of hyphal morphology must move from original assumptions of a steady-state system towards non-steady state assumptions of growth [34].

### Merging models with in vivo data

Mathematical descriptions of morphology and growth at the subcellular level are thus continuously refined. Do these models indeed form the conceptual basis for wet-lab data interpretation? In one recent example, conditional expression of the small GTPase encoding gene *arfA* in *A. niger* resulted in smaller pellet diameter in shake flask culture which occurred concomitantly with increased protein production [50]. These macromorphological effects were likely caused by decreased hyphal growth rate, reduced ability to maintain hyphal tip polarity, and a defective actin ring position at the hyphal tip due to altered *arfA* expression [50]. The actin ring has been shown to be the site of endocytosis in *A. nidulans*, which is maintained 1–2 µm behind the hyphal apex in this fungus and 2–3 µm behind the hyphal apex in *A. niger* [50, 51] (Fig. 1b). Geometric models of the spatial distribution of the actin ring in *A. nidulans* predict that this precisely maintained location ensures endocytic recycling of cell wall synthesising enzymes, cell end markers, and plasma membrane to maintain polarised growth and protein secretion at the tip [52]. In an *arfA* conditional expression strain of *A. niger*, fluorescent labelling of an actin binding protein revealed that the actin ring shifted approximately 1.2 μm towards the apex [50], which likely contributed to a loss of hyphal polarity, reduced hyphal tip growth and thus reduced pellet size. Modification of the actin ring location might thus represent a generic strategy for titrating morphology and enhancing protein secretion in industrial fungi [50]. Taken together, these studies demonstrate how increasingly sophisticated modelling of growth and morphology at macro and subcellular levels can lead to mechanistic explanations of fungal strain engineering in industrial settings.

## Tailoring growth and morphology to protein, acid, and secondary metabolite products: progress and knowledge gaps

Despite these advances in the fermentation control of fungal morphology and cognate modelling approaches, it is not currently possible to precisely predict the optimal morphology for a desired product and, consequently, it is necessary to invest significant efforts in process design. However, as the fundamental understanding and associated models of the filamentous lifecycle advances, it may be possible to use the underlying molecular, cellular, and developmental biology of fungi to predict improved growth and macromorphology for certain classes of product (i.e. acid, protein, or secondary metabolite).

### Protein secretion: tips, septa, and unconventional secretion pathways

Growth and protein secretion are coupled at the hyphal tip, whereby vesicles packed with cell wall synthesising enzymes and secretory proteins arise from the Golgi by budding [53], and subsequently travel along microtubules and actin filaments to the extending hyphal apex [20–22], aggregate in the Spitzenkörper, and become tethered to the plasma membrane by the exocyst [54], thus releasing vesicle cargo (Fig. 1b). Consequently, protein secretion is generally highest during rapid hyphal extension and periods of active growth (Fig. 3b). A growing body of evidence suggests that modifying fungal macromorphology for a maximum tip: biomass ratio is a useful approach for improving protein secretion in many fungal systems [55–57].

Interestingly, however, in some cases elevated hyphal tip number is not correlated with increased protein titre, which may suggest routes other than the tip are important in some instances. One recent explanation for the discrepancies between elevated hyphal tip numbers and titres of extracellular proteins is that unconventional protein secretion (UPS) pathways may also play important roles during industrial fermentation [58]. Generally, in UPS, proteins do not pass through the classical Golgi-vesicle-apex-dependent route, but are transported to the cell membrane via as yet undefined alternative mechanisms. Indeed, numerous extracellular proteins do not contain N-terminal signal peptides necessary for processing through the Golgi and packaging into extracellular vesicles, and consequently are predicted to be secreted via UPS [58].

A second possible complication in the relationship between hyphal tip number and extracellular protein titres is recent work suggesting that protein secretion may also occur at the hyphal septum. For example, in *A. oryzae* exocytosis and secretion also occur at intercalary hyphal regions (Fig. 1b), specifically at septal junctions [59, 60]. Secretion at hyphal septa plays a fundamental role in branch initiation and thickening of the cell wall at sub-apical locations and, in *A. oryzae*, the industrially relevant alpha-amylase was demonstrated to be secreted into the septal periplasmic space by fluorescent tagging [59]. In *A. niger*, growth on solid media with sugar beet pulp as a carbon source resulted in protein secretion both at the colony periphery and internal regions [61]. Fluorescent monitoring of the major secreted and industrially fermented glucoamylase protein in *A. niger* also demonstrates that this protein localises to intercalary hyphal regions, including septa [50, 62]. These data support the hypothesis that septal secretion could be of industrial relevance, and it is interesting to speculate that optimising morphology to maximise septal junctions through strain engineering efforts may be a future avenue to enhance product titres. Taken together, while several studies support the hypothesis that optimising fungal morphology by increasing hyphal tip numbers is a promising strategy to enhance protein production, both UPS and intercalary secretion pathways represent promising, yet underexplored, avenues for strain engineering efforts.

### Acids and secondary metabolites: a complex puzzle

For production of secondary metabolites and acids, predicting an optimal macromorphology based on mechanistic explanations of production and secretion/export is also problematic. For acid production, specifically citric acid in *A. niger*, several studies suggest that elevated titres occur with shorter hyphae or hyperbranched phenotypes [63, 64]. An exciting and important piece of the puzzle that has recently been revealed is the identification of the CexA major facilitator superfamily transporter that is required for export of citrate from *A. niger* [65]. However, this protein has yet to be localised to precise positions in the hyphal plasma membrane (e.g. tip, septa, or elsewhere), and consequently defining an optimal morphology to maximise CexA transporters for each mycelial compartment is currently challenging. Despite these limitations to our fundamental knowledge, however, it is clear that production of citrate occurs at specific stages of active hyphal growth. For example, recent dynamic modelling approaches have demonstrated that both oxalic and citric acid syntheses in *A. niger* occur following a diauxic switch to phosphate-limited growth [66] (Fig. 3b).

With regard to biosynthesis of secondary metabolites, a small pelleted morphology has been demonstrated to increase product titres in some instances, for example lovastatin fermentation by *A. terreus* [67]. Nevertheless, the underlying metabolic, molecular, and/or cellular basis for this improvement is currently unclear. What is certain, however, is that the formation of fungal secondary metabolites mostly occurs during periods of extremely low, or zero growth (Fig. 3b), which is due to the complex functions of these diverse bioactive molecules in the natural niche [68, 69]. Thus, an optimal morphology for secondary metabolite biosynthesis, in contrast to protein production, must somehow be integrated with nutrient limitation, thus causing ultralow fungal growth. A possible avenue for this is to generate pelleted morphologies with densely compact core, which may limit nutrient and oxygen diffusion and thus growth at the colony centre, in turn activating secondary metabolism [29]. Export of fungal secondary metabolites is also an extremely complex puzzle. Fungal natural products are biosynthesised by physically linked contiguous gene clusters, many of which contain genes encoding putative transporters that are predicted to be involved in extracellular secretion of the respective natural product [3]. Intriguingly, functional analyses of transporter genes in mycotoxin encoding clusters demonstrate that some of these transporters are functionally redundant, as deletion causes no reduction in secondary metabolite secretion [70]. Interestingly, in the model organism *A. nidulans*, deletion of a gene encoding a multidrug-resistant ATP binding cassette (ABC) transporter (which was physically located outside any predicted biosynthetic gene cluster) drastically reduced penicillin secretion [71], supporting the hypothesis that generic transporters could be used to maximise secretion of useful metabolites. Critically, determining the exact distribution of these transporters throughout the fungal cell or colony could enable rational design of morphology for maximum secretion of these molecules.

In summary, a complex relationship between fungal growth, morphology and protein, acid, and secondary metabolite titres emerges. Clearly, an optimal morphology will differ depending on the desired product, and despite significant knowledge gaps in the underlying mechanistic basis of product formation and secretion/export, it is now possible to postulate several generic morphological attributes or growth stages that may enhance fermentation efficiency in each case. Strain engineering efforts, increasingly informed by omics datasets, promise to deliver both the lead genes and platform strains for optimisation of filamentous morphology during diverse industrial applications.

## Rational strain engineering: Unlocking lead genes for optimised morphology and productivity from omics data

### Mutagenesis, comparative genomics, and functional genomics

Initial strain engineering efforts for optimal morphologies began in the 1950s, whereby industrial fungal isolates were mutagenized for improved biotechnological applications [72]. For a diverse range of fungi, strains displaying modified morphology following mutagenesis screens have generated elevated product titres and improved hydrodynamic performance in bioreactors. For example, UV and nitrous acid mutagenesis resulted in several hyperbranched *A. oryzae* strains causing less viscous culture broth during bioreactor cultivations but elevated glucoamylase production [73]. Elsewhere, diethyl sulfite mutagenesis of *T. reesei* generated a strain with short, highly branched hyphae that secreted over 60% more cellulase than the progenitor isolate [57]. The genomes of these production strain lineages are currently being sequenced in community-wide efforts to identify candidate genes for strain improvement from comparative genomic approaches to identify desirable properties with respect to morphology and hyperproductivity [15]. At present, however, studies which attempt to identify single nucleotide polymorphisms (SNPs) responsible for advantageous growth or production phenotypes in production strain lineages are limited. One such example used comparative genomics between the high protein producing industrial *A. niger* strain SH2, and progenitor isolate CBS 513.88 [74]. The hypersecretion phenotype of isolate SH2 is thought to be at least partially attributed to the highly branched hyphal fragments produced by this strain in submerged culture [74]. Comparative genomics between this strain and CBS 513.88 suggested that the mutant morphology may be explained by SNPs in genes encoding proteins that regulate or are necessary for cell wall synthesis, including components of the wall integrity pathway, chitin synthesis, and β-1,3-glucan synthesis [74]. Confirmation of these hypotheses, however, would require gene functional characterisation, and given that SNPs in several genes may synergistically contribute to the SH2 morphology, such wet-lab verification would require highly labour-intensive generation of combinations of *A. niger* mutants. Thus, while the genes identified from this comparative genomic study remain high priority candidates for engineering filamentous fungi for optimal industrial growth [74], their exact application in biotechnology remains to be determined.

Elsewhere, interrogation of UV mutagenized penicillin platform isolates of *P. chrysogenum* by comparative genomics has revealed repeated SNPs in a gene encoding a putative methyltransferase LaeA, which may concomitantly explain both elevated titres of beta-lactam biosynthesis and optimal morphology for improved rheology during submerged culture in these strains [75]. LaeA is a component of the heterotrimeric velvet complex in filamentous fungi [76] that was originally discovered in *A. nidulans* [77, 78]. The velvet complex consists of VeA, which is predominantly expressed in the dark and physically interacts with the protein VelB, which is expressed during hyphal growth and development [76]. VeA bridges VelB to LaeA, which in turn is hypothesised to reverse the formation of transcriptionally silent heterochromatin by DNA or H3K9 methylation activity [79], thus activating secondary metabolite gene loci during hyphal growth. The velvet complex is, therefore, a molecular nexus connecting light responses, hyphal growth, and secondary metabolism. LaeA mutants have been generated in numerous fungal cell factories, which have been used to concomitantly activate natural product formation and modify morphology in many [75, 80, 81] but not all species [82]. Thus, the LaeA encoding gene is a useful example to highlight how generic strain improvement strategies, originally identified from mutagenesis and/or comparative genomic approaches, can be used to control differentiation and production of useful metabolites in various fungal species. It is likely that other such key regulators of development (e.g. StuA, FlbA, BrlA) might soon be common components of the biotechnologist’s toolkit to activate or improve natural product formation in industrial fungi [3, 83].

Clearly, comparative genomics is a powerful approach to unlock lead genes from mutagenized isolates for strain improvement programs. A recent experimental technique developed in *A. niger*, termed bulk segregant analysis, enables the precise mapping of an SNP with the corresponding phenotype and, thus, may compliment in silico analysis of mutagenized genomes [81]. This approach requires a sexual or parasexual cycle in the fungus of interest, as the mutagenized isolate is firstly crossed with a wild-type strain. Next, haploid segregants displaying the phenotype of interest are DNA sequenced in order to identify SNPs [81]. Importantly, the SNP absent in the progenitor strain, and concomitantly present in all segregants, is responsible for the mutant phenotype. In a proof of principle experiment, the developers of this technique analysed a non-acidifying phenotype of a UV-mutated *A. niger* isolate [81]. Following bulk segregant profiling, it was confirmed that an SNP in a gene-encoding LaeA was responsible for *A. niger* non-acidification, thus further linking chromatin remodelling, and development with product titres. Thus, bulk segregant analysis is a powerful approach which could in future be applied to conclusively reverse engineer the SNPs, and encoding genes, that result in biotechnologically advantageous growth and morphology from libraries of mutagenized fungal isolates.

### Transcriptomics

**Fig. 4:**
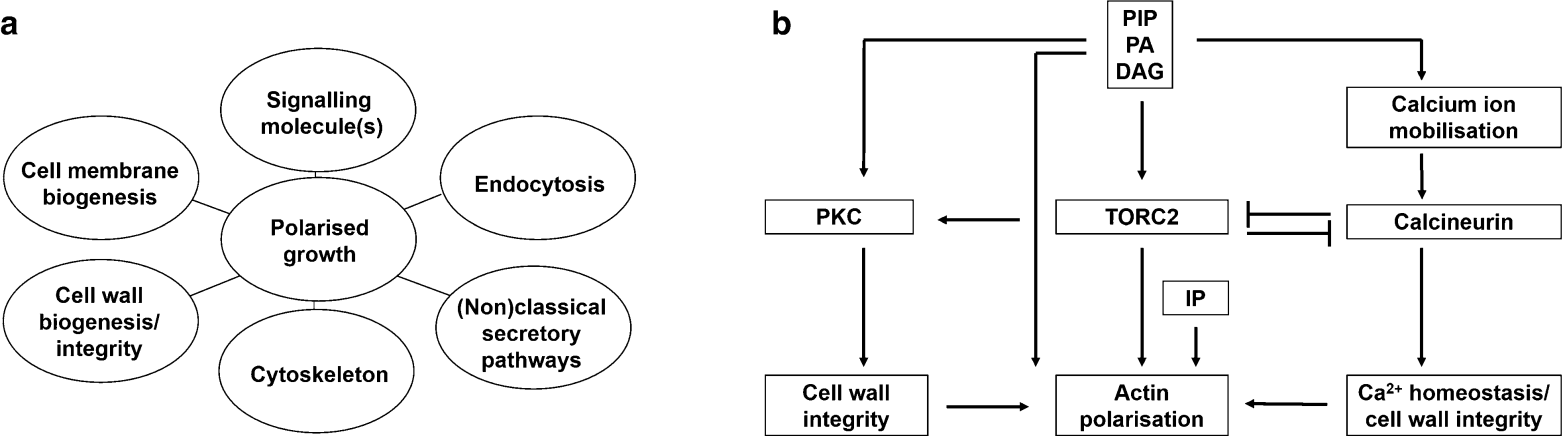
Cellular processes that are essential for morphogenesis in filamentous fungi as deduced from transcriptomic studies. **a** Genes belonging to various key processes are transcriptionally deployed during submerged fungal growth in multiple species. Note, for each fungal species, it is common for several hundred differentially expressed genes to belong to each cohort. **b** Proposed gene-network controlling polarised growth and branching in *A. niger* [24]. (Phospho)lipid signalling molecules including phosphatidylinositol phosphates (PIP), phosphatidic acid (PA) and diaclyglycerol (DAG) are supposed to regulate the activity of the TORC2 complex, the activity of the protein kinase C (PKC) and might mobilise calcium from internal stores. TORC2 signalling likely plays a crucial role in maintaining polarity by directly controlling actin polarisation but also by inhibiting calcineurin signalling. TORC2 is also essential for cell wall biosynthesis due to activation of PKC, which is the initiating kinase of the cell wall integrity (CWI) pathway. Inositolphosphate (IP) is also proposed to control actin polarisation. For details, see [24]

In addition to genomics approaches, RNA seq and microarray gene expression profiling during experimental models of enzyme, acid, and natural product fermentation have revealed potential gene candidates for optimising fungal morphology across diverse industrial processes. Various experimental designs have been utilised, for example, time-series analysis throughout *A. niger* citric acid fermentation [63], comparisons between low and high penicillin producing isolates of *P. chrysogenum* [84], during over-expression of the glucoamylase encoding gene in *A. niger* [85] and during bioreactor cultivation of wild-type and hyperbranching *A. niger* strains [24, 86, 87]. It is clear that genes belonging to common morphology and growth-associated processes are transcriptionally deployed, including classical and non-classical secretory pathways, cytoskeleton components, endocytosis, exocytosis, cell wall and cell membrane biosynthesis (Fig. 4a). Including the various signalling pathways driving and controlling these subcellular processes, it has been estimated that as many as 2000 genes encode proteins that at a certain level may participate in filamentous fungal growth and development [63, 84, 85, 88].

As just one example, the *A. niger* chitin synthase encoding gene An12g10380 (*chsE*) is transcriptionally upregulated during citrate fermentation [63] and following over-expression of a glucoamylase encoding gene [85], strongly suggesting that (i) chitin synthesis at the cell wall is a critical component of morphological development during industrial applications, and (ii) genetic targeting of this process could be used to modify and possibly optimise morphology. This hypothesis has been validated by RNAi knockdown of chitin synthase encoding genes in *A. niger* (*chsC*) and *P. chrysogenum* (*chs4*), which result in compact pellets and highly branched morphology, and eventually in elevated citric acid (40%) and penicillin product titres (27–41%), respectively [89, 90].

More generally, numerous transcriptional studies support the hypothesis that diverse cell signalling networks orchestrate growth, morphology, and development in multiple filamentous cell factories [24, 63, 84–88]. Signalling cascades are interconnected networks that transduce extracellular environmental signals into cellular responses, including, for example, nutrient availability, cell wall integrity in response to sheer stress, and osmotic perturbation (see next section for detail) [91]. Based on transcriptomics signatures, a signalling network controlling morphogenesis was reconstructed for *A. niger* in 2009 and refined in 2013 [24, 86, 87]. It has been hypothesized that phospholipid signalling, sphingolipid signalling, target of rapamycin kinase (TORC2) signalling, calcium signalling and cell wall integrity (CWI) signalling pathways concertedly act to control polar growth in *A. niger* (Fig. 4b). The reconstructed transcriptomic network model obtained implies that these pathways become integrated to control sterol, ion transport, amino acid metabolism and protein trafficking to ensure cell membrane and cell wall expansion during hyphal growth. Most importantly, this transcriptomic network predicted that the transcription factors RlmA, CrzA and at least a third, so far unknown, transcription factor are output genes of the CWI signalling pathway. This was subsequently experimentally confirmed by identification of the transcription factor MsnA which—at least in *A. niger*—not only controls osmotic stress but is also responsible to ensure cell wall integrity under cell wall stress conditions [92].

A final example for the successful deduction of lead genes from transcriptomic data for improved morphology and productivity is the Rho GTPase RacA, which was hypothesized to control filamentous growth via actin polymerisation and depolymerisation at the hyphal apex in *A. niger* [93]. Transcriptional profiling of a *racA* deletion and dominant activation allele suggested that this protein plays a critical role in morphology and protein secretion [87] and that deletion of *racA* in *A. niger* results in a hyperbranched phenotype. Subsequent gene functional studies revealed that concomitant overexpression of the glucoamylase encoding *glaA* gene in this background using the metabolism-independent gene switch Tet-on [94] enables a 400% increase in glucoamylase secretion [55]. Given that *racA* is highly conserved in filamentous fungi [17], it is possible that *racA* mutant isolates could be widely applied to enhance protein secretion in other systems, including *Trichoderma* spp., *Penicillium* spp., and others.

### Genome wide metabolic models

Genome wide metabolic models (GWMM) of various fungal cell factories have recently been developed and offer novel avenues to accurately predict gene knockout phenotypes or maximum product yields under different nutritional sources. The ultimate aim of GWMM is to predict most of the metabolite content of an organism and link these with cognate reactions and catalytic enzymes. Arguably, the best such model in the fungal kingdom is for the budding yeast *Saccharomyces cerevisiae*, which contains over 1400 metabolites, 1800 biochemical reactions, and 900 genes encoding the catalysing enzymes [95]. These models have enabled sophisticated predictions of protein function related to fungal growth, for example regulation of acetyl-COA biosynthesis by the Oaf1 transcription factor encoding gene in yeast [96]. GWMMs for numerous filamentous cell factories have been developed over the last decade [97–99] and have been used to model conditions for maximum production of fermentation products, for example secreted proteins in *A. oryzae* [100] and *A. niger* [101]. More recently, strain-specific models have been updated, for example in *A. niger*, with information from several hundred publications curated to add 770 metabolites, 940 reactions, and 454 genes [102]. Integration of these GWMM into publicly available data repositories including FungiDB [17], MycoCosm [15] and Ensembl [103] promises to facilitate numerous avenues towards improved growth, nutrient utilisation, activation of secondary metabolism, and other diverse applications in subsequent strain engineering experimentation [1]. While currently linking metabolism and filamentous morphology is challenging, these public models will likely be critical for future hypothesis generation. Specifically, finding bottlenecks that sustain/fuel anabolic processes, which themselves are prerequisites for the maintenance of hyphal growth, could eventually become important candidate genes for engineering morphology.

In summary, comparative genomics, transcriptomics, and metabolic models have identified hundreds, or even thousands of genes that are promising candidates for engineering morphology in industrial fungi. This work, combined with numerous gene functional characterisation experiments in industrial and model fungi, has identified what is arguably one of the most promising strain engineering strategies for controlling growth and morphology: genetic targeting of fungal signalling cascades. The next section introduces some key aspects of fungal signal transduction and highlights how these are currently being rationally manipulated for optimised industrial applications.

## Targeting signalling cascades for modifying polar growth and morphology in industrial applications

**Fig. 5:**
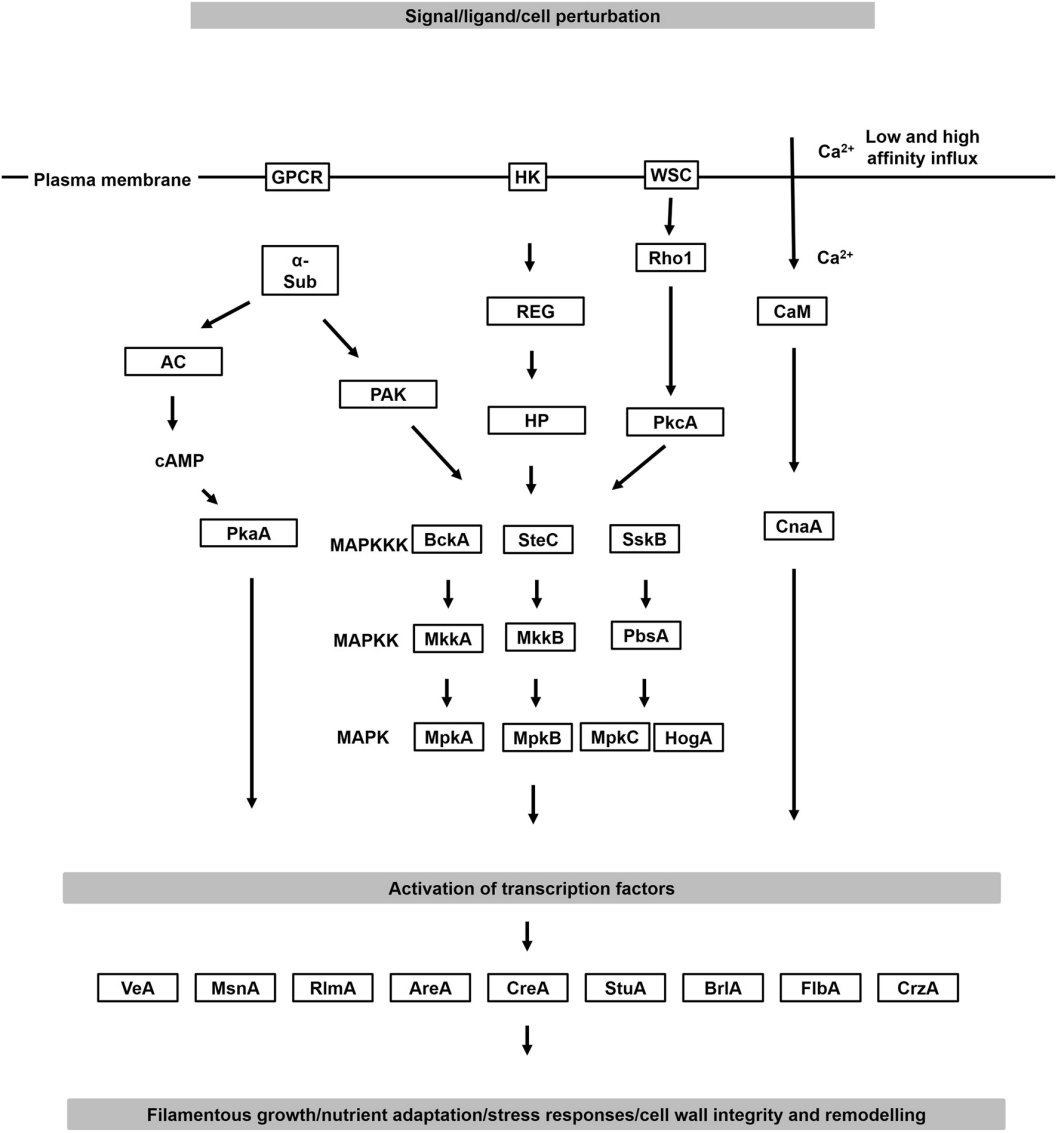
Simplified schematic depiction of the major signalling cascades in filamentous fungal cell factories. MAPK cascades are initiated at the plasma membrane by two main processes. Firstly, a G protein’s α subunit activates a protein activated kinase (PAK), which phosphorylates an MAPKKK. Secondly, in the two-component signal transduction system, a transmembrane histidine kinase (HK) is activated by extracellular ligands and a response regulator (REG) activates a histidine-containing phospho-transmitter (HP) that subsequently activates MAPK signalling. Alternatively, mechanosensors such as WSC receptors [104] at the cell surface are activated by cell wall perturbation, which activate MAPK cascades via GTPases (e.g. Rho1) and protein kinase C (PkcA). Once active, a phosphorelay system between MAPKKK, MAPKK and MAPK results in phosphorylation of downstream transcription factors. In the PKA/cAMP pathway, a G-protein coupled receptor (GPCR) is activated at the plasma membrane and ultimately the G protein’s α subunit (α-sub) dissociates from the GPCR complex and activates an adenylyl cyclase (AC). This, in turn, catalyses the conversion of ATP into cAMP. Increases in concentration of the second messenger cAMP activates protein kinase A (PKA), which phosphorylates various target proteins, including transcription factors. These enter the nucleus and regulate diverse responses. In calcium signalling, low- and high-affinity Ca^2+^ influx systems are activated at the plasma membrane. Ca^2+^ ions bind and activate calmodulin (CaM), which in turn binds to subunit A of the protein calcineurin (CnaA). Once activated, calcineurin dephosphorylates the transcription factor CrzA, which causes elevated expression of genes necessary for growth and diverse stress responses. Depicted are exemplar transcription factors that regulate filamentous growth (BrlA, StuA, FlbA, CrzA), cell wall integrity (CrzA, MsnA, RlmA), adaption to carbon limitation (CreA) and nitrogen limitation (AreA). All pathways have critical control of filamentous growth, fungal morphology, and development. Gene names are taken from *A. niger* or the model organism *A. nidulans*. Note that extensive cross talk occurs between pathways, and that in this schematic not all possible membrane receptors, signalling proteins, or transcription factors are depicted. Interested readers are guided to excellent reviews which cover fungal signalling cascades in greater depth ([91, 118])

Given the crucial role that cell signalling plays in regulating morphology, numerous strain engineering efforts have targeted components of these cascades to optimise growth for improved biotechnological applications. In filamentous fungi, the major signalling pathways include mitogen activated protein kinase (MAPK) cascades, protein kinase A (PKA)/cyclic adenosine monophosphate (cAMP) signalling, and calcium ion responses (Fig. 5), all of which are, arguably, promising targets for strain engineering approaches to optimise morphology and growth of different industrial fungi. Selected examples will be discussed in the following section.

### MAPK signalling pathways

MAPK cascades are initiated at the plasma membrane by G-protein coupled receptor (GPCR), a transmembrane histidine kinase or so-called WSC receptors ([104], Fig. 5). A phosphorelay system between an MAPK kinase kinase, MAPK kinase, and MAPK results in the phosphorylation and regulation of chromatin remodelling proteins, transcription factors, and co-regulatory proteins which activate and/or repress gene expression in the nucleus. Three MAPK signalling cascades have been described in filamentous fungi, which regulate filamentous growth and spore formation in response to pheromone and nutrient availability (MpkB cascade), environmental adaptation to oxidative and osmotic stress responses (MpkC/SakA/HogA cascade), and cell wall integrity pathway in response to cell wall perturbation (MpkA cascade, Fig. 5) [91].

In several instances, MAPK phosphorylation of downstream transcription factors that control filamentous growth and development have been identified, mainly in the model organism *A. nidulans*. For instance, the MpkB controls the regulator SteA, which concomitantly induces sexual development and inhibits the activation of transcription factor MedA, which is also involved in conidiophore and sexual development (reviewed in [105]). Also in *A. nidulans*, MpkB interacts with the conserved nuclear transcription factor SteB and regulatory velvet protein VeA, which are necessary for initiation of (a)sexual development and coordination of secondary metabolite production, respectively [106]. Consequently, deletion, overexpression or RNAi-based knock down of various levels of MAPK signalling cascades can cause diverse changes in morphology in filamentous fungi that may be biotechnologically advantageous, including hyperbranching (e.g. following deletion of the MAPKKK *steC in A. nidulans*) [107], loss of conidiation (e.g. following deletion of a MAPKK encoding gene *mkkB* in *A. niger*) [108], and enhanced growth rate (e.g. following deletion of the MpkB orthologue in *T. reesei*) [109]. Despite the pleiotropic consequences of genetic targeting of MAPK signalling cascades, recent work has demonstrated that they can be used in rational strain engineering efforts. In a proof of principle experiment, deletion of the gene predicted to encode an MkpB orthologue in *T. reesei* resulted in elevated growth rates and consequently increased production of cellulases during submerged growth [109]. It remains to be determined how strain engineering of other components of MAPK signalling can be applied in other species.

### The cAMP/PKA signalling pathway

cAMP/PKA signalling regulates vegetative growth, carbon sensing, and other environmental conditions such as light [105]. In this pathway, activation of a GPCR causes an adenylate cyclase to catalyse the conversion of ATP into cAMP, which subsequently activates cAMP-dependent protein kinase A (PKA). The activated PKA phosphorylates various target proteins, including transcription factors, resulting in their entry to the nucleus and modification of gene expression (Fig. 5). In concordance with the vital role of the cAMP/PKA pathway on filamentous growth, deletion of various components can be used to modify morphology, including the adenylate cyclase and PKA encoding genes [110, 111]. In *T. reesei*, ACY1 and PKAC1 genes co-ordinate light, filamentous growth, and cellulase gene expression, offering an avenue to concomitantly titrate morphology and cellulase expression [111]. In addition to deletion, overexpression of PKA signalling can be used as a strategy to modify fungal macromorphology. For instance, in *A. niger*, overexpression of the PKA subunit PkaC resulted in a more compact colony morphology [112]. Interestingly, in addition to regulating growth and morphology, the cAMP/PKA pathway also controls fungal secondary metabolism. For example, in the model organism *A. nidulans,* a dominant activating allele of a gene encoding the alpha-subunit of a heterotrimeric G-protein, *fadA*, resulted in elevated transcription of genes from the penicillin gene cluster, higher titres of penicillin production, and reduced conidiation [113]. Thus, the cAMP/PKA signalling pathway is of interest with regard to controlling fungal morphology while concomitantly modifying the expression of natural product biosynthetic genes.

### The calcium/calcineurin signalling pathway

The calcium/calcineurin pathway has been extensively studied as a potential drug target in fungal pathogens of humans, where it regulates growth, morphology, stress responses and virulence [114]. Specifically, cell stress activates low and high-affinity Ca^2+^ influx systems at the plasma membrane after which Ca^2+^ ions bind and activate the cytosolic protein calmodulin, which in turn binds to subunit A of the protein calcineurin (Fig. 5). Once activated, calcineurin dephosphorylates the transcription factor CrzA, which causes elevated expression of genes necessary for growth and diverse stress responses [115]. The calcineurin signalling pathway is an important regulator of asexual growth, for example in *Aspergillus* spp., where CrzA mediates developmental induction via the transcription factor BrlA [116]. In *T. reesei* deletion of the CrzA encoding gene caused a hyperbranched phenotype which was paralleled with elevated secretion of hemi-cellulases [117]. Moreover, CrzA is necessary for responses to withstand cell wall stress encountered during high bioreactor stir speeds, and this pathway is required for elevated chitin, glucan and cell wall protein levels in *A. niger* and *T. reesei* as the cell wall is reinforced [92, 117]. Thus, the calcium/calcineurin pathway and transcription factor CrzA are promising targets for biotechnological manipulation of fungal growth, development, and stress resistance.

### Further signalling pathways

In addition to these main signalling mechanisms, there are numerous other signal transduction pathways in filamentous fungi that regulate morphology, growth and development, including responses to pH (via membrane receptor PalH and transcription factor PacC), light (via the velvet complex, see above), additional nutrient sensing pathways (via the target of rapamycin protein kinase TORC2), response to reactive oxygen species (via transmembrane NADPH oxidases), and RAS signalling [91, 118]. Given that all of these pathways transduce extracellular signals to regulate interconnected and diverse aspects of morphology and development, they are also promising targets for strain engineering. It remains to be seen if the pleiotropic consequences of genetic manipulation of these pathways are advantageous, or a limitation for strain engineering of industrial fungi. One example of the limitations to this strategy involves the heterotrimeric velvet complex (Fig. 5). In *T. reesei*, deletion of a gene encoding the velvet protein Vel1 (the orthologue of *A. nidulans* VeA) resulted in a hyperbranched phenotype, but a complete inhibition of cellulase and xylanase expression [119, 120]. These studies highlight potential pitfalls of manipulating signalling cascades and proteins that are components of the complex and dynamic architecture for fungal environmental sensing and adaptation. A long-term goal for maximum control of fungal morphology during industrial applications may thus be to develop strains with reduced genome complexity. We thus discuss several recent technological developments in the field of fungal synthetic biology below.

## Synthetic biology, genome engineering and beyond

As stated above, thousands of genes may contribute to the complex phenotype of fungal morphology. This complexity results in emergent properties that cannot currently be predicted or explained based on understanding of the constituent components [121]. In this regard, the revolutions in the field of synthetic biology promise to deliver the next generation of filamentous cell factories by delivering chassis cells that contain either designer chromosomes, or minimal genomes, with drastically reduced complexity and thus improved engineering capabilities.

Progress towards a minimalized fungal genome has moved at a rapid pace in the unicellular yeast *S. cerevisiae*. In 2011, Dymond and colleagues synthesised a reduced version of the budding yeast chromosome 3, lacking ~ 14% of wild-type base pairs, with tRNA and transposons removed [122]. Remarkably, the 16 *S. cerevisiae* chromosomes have been reduced by genome editing and fusion experiments, and viable strains with two or even a single chromosome have been generated [123, 124]. Although much less advanced than in *S. cerevisiae*, a technology for filamentous fungal genome minimalization has recently been demonstrated in *A. niger* [125]. In this study, low targeting of exogenous cassettes in recipient genomes was obviated by inactivation of the non-homologous end joining pathway, after which individual genes or large (~ 48 kb) sections of chromosomes were deleted using CRISPR–Cas9 [125]. In a proof of principle experiment, a cluster necessary for the biosynthesis of the mycotoxin fumonisin was removed. Similar CRISPR–Cas9 gene editing systems are now available for *P. chrysogenum* [126], *T. reesei* [127], *A. oryzae* [128], *M. thermophila* [129] and other filamentous fungal species harnessed in industrial applications. While the gene content of filamentous fungi is considerably higher than that of yeast (e.g. *A. niger* ~ 14,000; yeast ~ 6000), and the number of experimentally verified essential genes considerably less [130, 131], the fundamental tools and proof of principle for genome minimalization have now been developed [123–125]. Thus, it is conceivable that minimal genomes exclusively containing the necessary genes required for a user-defined growth phenotype or morphology could be developed in the future.

Other than CRISPR–Cas, what other synthetic tools and techniques promise to revolutionise fungal cell factories, both from morphological perspectives and for increasing the associated product portfolio? Several filamentous fungi have been engineered to heterologously express key natural product biosynthetic genes, such as those encoding nonribosomal peptide synthetases, or polyketide synthases, including *A. nidulans* [132, 133], *A. oryzae* [134], *A. niger* [6], and *P. chrysogenum* [135], amongst others. Excitingly, new-to-nature compounds can also be generated, either by swapping of enzyme domains, subunits, or modules [136, 137], or by feeding various amino acid precursors in growth media, which are incorporated into nonribosomal peptide molecules [6]. Thus, in future, fungal cell factories can not only be optimised for improved morphology, but also to heterologously express high-value products including new-to-nature compounds.

Further synthetic biological advances are complimentary to the above natural product discovery pipelines. This includes, for example, the development of polycistronic gene expression approaches in filamentous fungi [138–140]. Given that transcriptomic analyses reveal highly coordinated and stage-specific transcriptional deployment of gene cohorts throughout growth in industrial applications [63, 85, 141], the capability of concomitantly controlling the expression of multiple morphological regulatory genes using a single promoter may offer an attractive solution for improved morphological engineering studies.

A further important conceptual point with regard to engineering morphology, revealed from the use of the synthetic Tet-on gene switch in *A. niger* [50], is that null or constitutive overexpression approaches may not be sufficiently precise genetic manipulations to reveal or control industrially relevant morphology phenotypes in platform strain development pipelines. For example, transcriptional profiling during carbon-dependent enhancement of protein secretion in *A. niger* revealed that the putative *arfA* GTPase encoding gene was upregulated by only a moderate amount under these conditions (i.e. 30%) [141]. Subsequent functional analysis of this gene by replacement of the native promoter with the tunable Tet-on gene switch revealed that it is essential, and, moreover, that distinct morphologies and protein production phenotypes were revealed from titratable control of *arfA* expression [50]. Consequently, conditional and tuneable synthetic gene switches which are functional in filamentous fungi and have gone through multiple rounds of engineering and optimisation [94, 142, 143] represent an attractive tool that offers more precise interrogation of the relationship between gene function and strain morphology when compared to classical deletion or constitutive over-expression approaches.

These molecular advances have occurred concomitantly with developments in fungal imaging. For example, three‐dimensional morphology of *A. niger* and *P. chrysogenum* pellets has most recently been quantified by X‐ray microtomography [144]. Excitingly, this technology opens up new avenues for accurately quantifying hyphal distributions in the pellet core, including hyphal density, hyphal branch rates, and tip numbers. Thus, future studies on pellet morphology can now begin to access how different pellet phenotypes impact product titres.

In summary, these technological advances highlight how many synthetic biological tools are already optimised for filamentous fungi. We predict that these will enable the development of new cell factories with optimised morphologies, minimalised genomes, and improved product formation based on precise gene transcriptional control.

## Conclusions

Advances in fundamental science and modelling approaches are beginning to reveal the molecular and cellular basis of product formation and secretion in filamentous fungi under industrial, i.e. bioreactor, conditions, and consequently rational design based on predictions of optimised morphology may increasingly be possible. A wealth of omics data is currently available and comparative analyses have already shown on how to unlock these data. Hence, targeted genetic manipulation of candidate genes controlling or indirectly impacting morphology can increasingly be used to generate and test novel strains for optimal growth. In parallel with these trends, fundamental progress in synthetic biology promises to reduce genome complexity of filamentous fungi, which ultimately may deliver chassis cells that have highly controlled and predictable growth and development for maximum product titres and enhanced performance in bioreactor cultivations. Hence, the technological tools are thus in place for data-driven strain improvement programs. Still, the insights generated so far also touch on some fundamental questions, which need to be addressed to fully exploit the potential of filamentous fungi for a sustainable bio-economy: from an evolutionary point of view, are multicellularity and polar growth a prerequisite for high protein secretion? Can the molecular basis of filamentous and multicellular growth be significantly reduced, or are too many of the components essential for high productivities? From a bioprocess engineering perspective, is it possible to develop a universal model of fungal growth, from dynamic changes in subcellular structures in young (un)branched hyphae to macroscopic units? Are generic solutions to engineering morphology and growth in the diverse repertoire of industrial filamentous fungi possible, or do deviations in gene and protein function make this goal unrealistic? As with the last decades, fundamental and applied sciences on filamentous fungi have to go hand in hand to mutually benefit from each other and to synergistically contribute to answering these questions.

## Abbreviations

ABC: ATP binding cassette
cAMP: cyclic adenosine monophosphate
CRISPR: clustered regularly interspaced short palindromic repeats
Cas: CRISPR-associated
CWI: cell wall integrity
GPCR: G-protein coupled receptor
GWMM: genome wide metabolic model
MAPK: mitogen activated protein kinase
MAPKK: mitogen activated protein kinase kinase
MAPKKK: mitogen activated protein kinase kinase kinase
PKA: protein kinase A
SNPs: single nucleotide polymorphisms
TORC2: target of rapamycin complex 2
UPS: unconventional protein secretion
